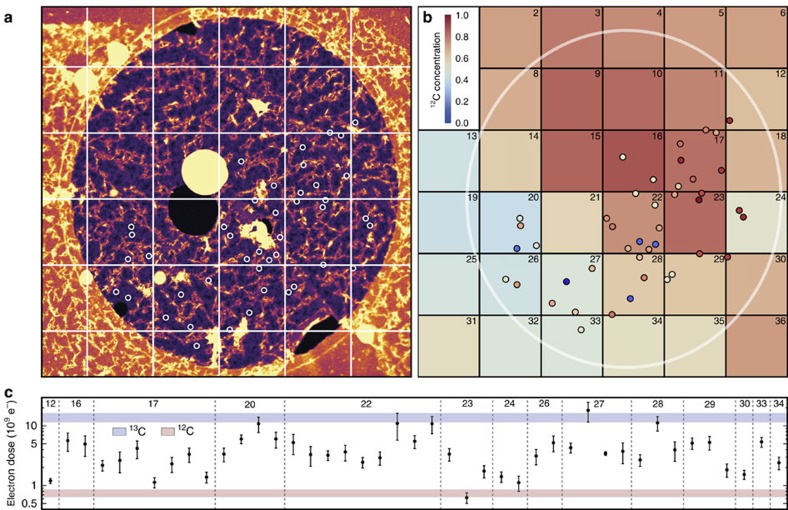# Corrigendum: Isotope analysis in the transmission electron microscope

**DOI:** 10.1038/ncomms15780

**Published:** 2017-08-30

**Authors:** Toma Susi, Christoph Hofer, Giacomo Argentero, Gregor T. Leuthner, Timothy J. Pennycook, Clemens Mangler, Jannik C. Meyer, Jani Kotakoski

Nature Communications 7: Article number: 13040; DOI: 10.1038/ncomms13040 (2016); Published: 10 10 2016; Updated: 08 30 2017

This Article contains typographical errors in Fig. 3 and Equation 23.

In Fig. 3c, the labels ‘^12^C’ and ‘^13^C’ in the key should be reversed. The correct version of Fig. 3 appears below as Fig. 1.

The correct form of Equation 23 is as follows:




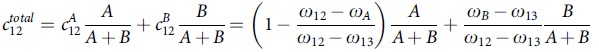




## Figures and Tables

**Figure 1:**