# Supplementary material for: Isotope analysis in the transmission electron microscope
Source: Nat Commun. 2016 Oct 10;7:13040. doi: 10.1038/ncomms13040 (PMC5476802; doi:10.1038/ncomms13040)
Supplement: Supplementary Information — Supplementary Figures 1-3 and Supplementary Table 1 [file ncomms13040-s1.pdf]

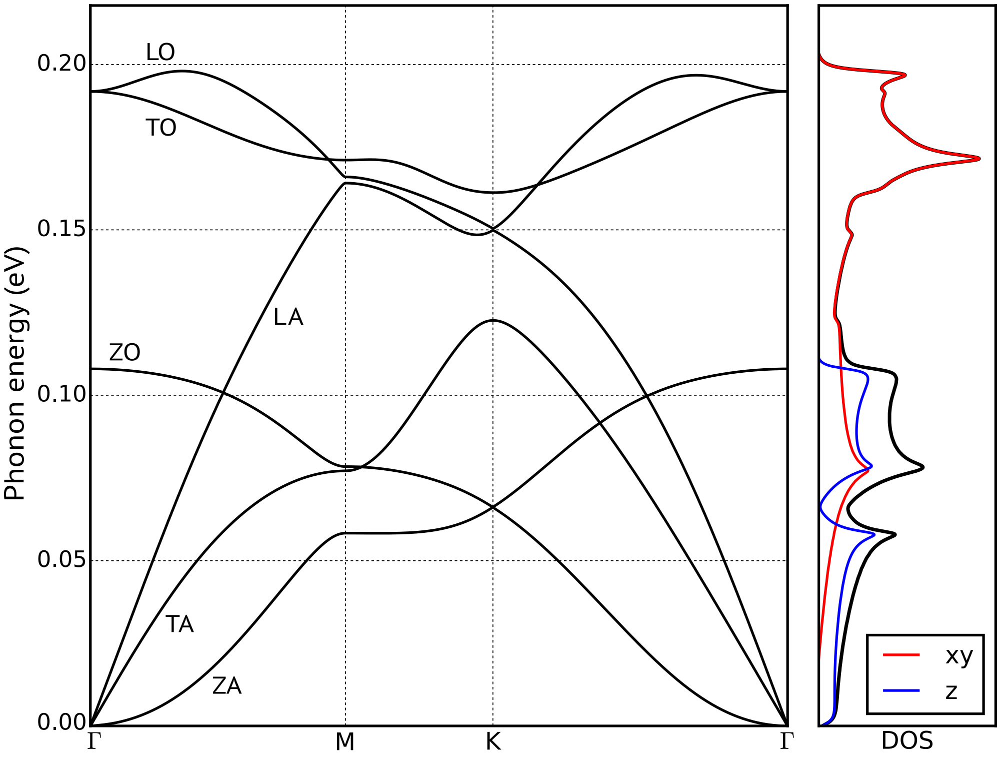

Supplementary Figure 1: The calculated graphene phonon band structure and the corresponding density of states. The out-of-plane component (z) is drawn in blue, in-plane (xy) one in red. Γ, M and K denote the symmetry points of the Brillouin zone, and the phonon branches are labeled with 'Z' = out-of-plane, 'T' = transverse, 'L' = longitudinal, 'A' = acoustic, 'O' = optical.

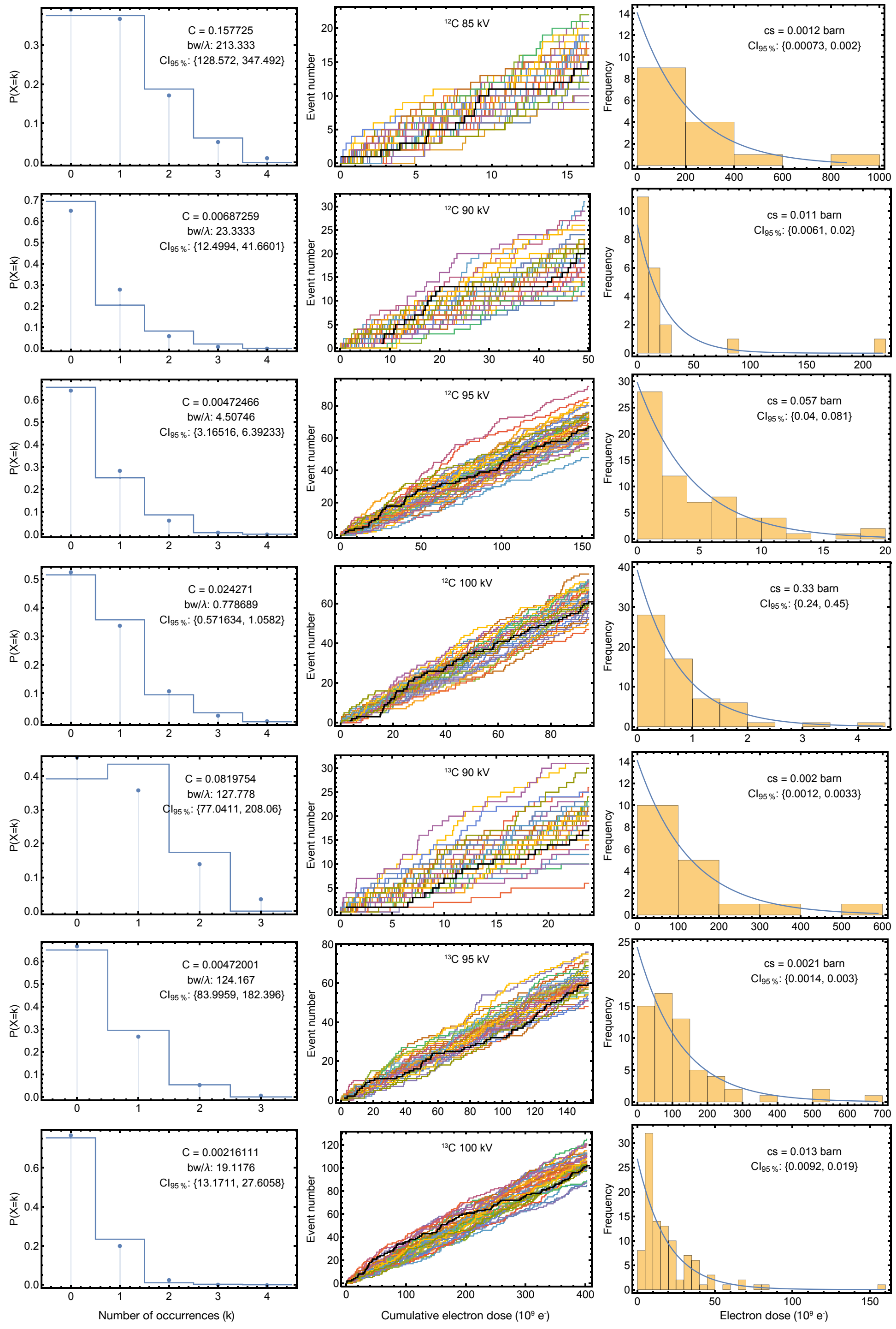

Supplementary Figure 2: Poisson analyses of the dose data. 'C' denotes the value of the Cash C-statistic calculated between the fitted Poisson process and the data, 'bw' the bin width, and 'cs' the resulting cross section.

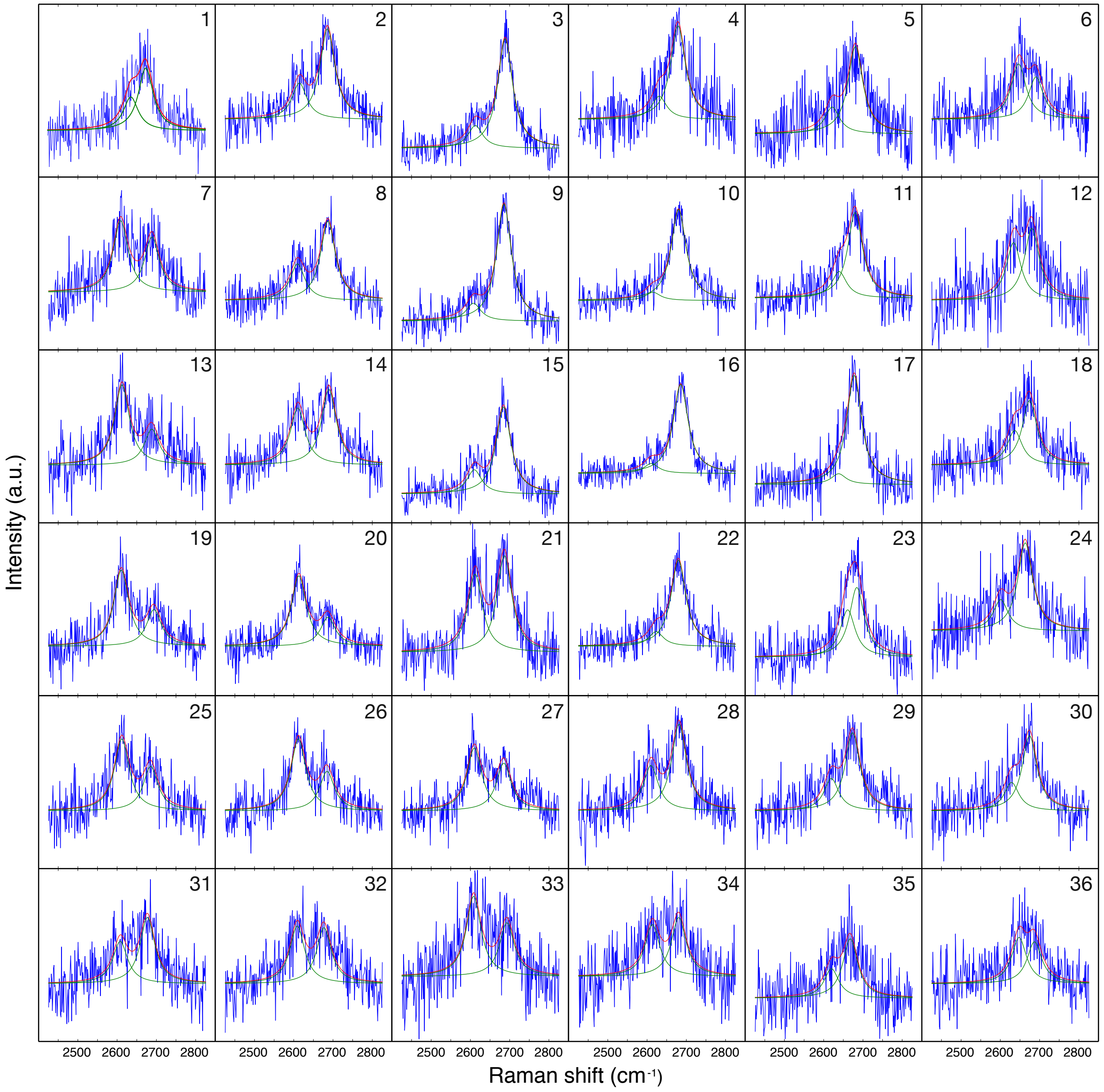

Supplementary Figure 3: The recorded and fitted Raman 2D spectra. The measured spectra are shown in blue, the fitted peaks in green, and their sum in red. The numbers at the top right corners of each panel correspond to the grid squares of Figure 3 of the main manuscript.

Supplementary Table 1: Parameters for determining the cross sections of article Figure 2. The columns show the number of measured STEM time series N, the average dose rate† $\dot{D}$ (in units of 10^8 e$^-$s^{-1}, with the lowest and highest rates given in brackets below), the expected Poisson dose λ (in units of 10^9 e$^-$) and the experimental (STEM) and predicted (DFT) cross sections σ_d and their standard errors (in millibarn) for each electron acceleration voltage U (in kV).

	^{12}C					^{13}C				
	STEM				DFT	STEM				DFT
U	N	$\dot{D}$	λ	σ_d		N	$\dot{D}$	λ	σ_d	
80	–	–	–	–	4×10^{-3}	–	–	–	–	7×10^{-6}
85	15	2.25 [1.93,2.56]	213.3	$1.2^{+0.8}_{-0.5}$	0.2	–	–	–	–	9×10^{-4}
90	21	1.62 [0.95,2.28]	23.3	$10.9^{+9.5}_{-4.8}$	5.3	18	2.43 [2.22,2.63]	127.8	$2.0^{+1.3}_{-0.8}$	5×10^{-2}
95	67	0.94	4.51	$56.6^{+24.0}_{-16.7}$	59.1	60	2.54 [2.26,2.82]	124.2	$2.1^{+1.0}_{-0.7}$	1.4
100	61	7.19 [6.44,7.94]	0.78	328^{+119}_{-87}	340.1	102	1.13 [1.01,1.24]	19.1	$13.3^{+6.0}_{-4.1}$	18.3

† To calculate the doses in Supplementary Data 2, we made a linear interpolation between the dose rates measured at the beginning and end of each experiment, and assigned a dose rate to each dataset according to its time. The ranges given in the table correspond to the minimum and maximum dose in any of the experiments comprising the dataset.
